# Puberty-Associated Gingival Enlargement Exacerbated by Polycystic Ovary Syndrome: A Case Report

**DOI:** 10.4317/jced.64044

**Published:** 2026-07-29

**Authors:** Nipun Dhalla, Lipika Gopal, Garima Asthana, Vinanti Rekhan, Pragya Goyal, Ruchi Pandey

**Affiliations:** 1 Department of Periodontology, Manav Rachna Dental College, SDS, Manav Rachna International Institute of Research And Studies, Haryana

## Abstract

**Background:**

Polycystic ovary syndrome (PCOS) is a common endocrinopathy affecting reproductive-aged women, characterised by metabolic abnormalities, polycystic ovaries, hyperandrogenism, and chronic anovulation. This pathology has been attributed to hormonal imbalances, including elevated levels of insulin, growth hormone (GH), ghrelin, LEAP-2, GnRH, LH/FSH ratio (>2:1), androgenic hormones, and estrogen. While puberty-associated gingival enlargement is primarily triggered by dental plaque, systemic hormonal fluctuations can significantly exacerbate the tissue response.

**Case Presentation:**

A 17-year-old female presented with spontaneous gingival bleeding and generalized gingival overgrowth persisting for one year. Clinical examination revealed fair oral hygiene and extensive inflammatory swelling of the marginal, interdental, and attached gingiva with bleeding upon probing, but no evident bone loss. Medical evaluation confirmed PCOS based on irregular menses, weight gain, acne, elevated serum testosterone, an increased LH/FSH ratio, hyperinsulinemia, and polycystic ovarian morphology. Initial management consisted of non-surgical periodontal therapy, including scaling, root planing, and oral hygiene instructions combined with gynaecological care for PCOS, which successfully reduced acute inflammation. Residual fibrotic enlargement was subsequently treated via surgical gingivectomy and gingivoplasty. Postoperative healing was uneventful, yielding satisfactory functional, aesthetic, and periodontal outcomes.

**Conclusions:**

This case highlights that PCOS may contribute to or exacerbate gingival enlargement through hormonal and inflammatory alterations rather than being a direct etiological factor. Early interdisciplinary diagnosis and co-management between dental and medical professionals are crucial for optimizing both periodontal and systemic health outcomes in adolescent patients.

## Introduction

Gingival hyperplasia is an increase in the number of cells within the gingival tissue. An abnormal tissue reaction to inflammation caused by local irritants such as calculus and plaque, as well as systemic disruptions such as hormone fluctuations, can result in gingival hyperplasia. The most common form arises from inflammation of the surrounding gingival tissues caused by dental plaque. In addition to plaque-induced inflammation, several other factors can trigger or worsen gingival overgrowth. Hormonal changes during puberty or pregnancy can alter the tissue's response to local irritants, resulting in more pronounced enlargement (1). Certain systemic medications also strongly contribute to drug-induced gingival overgrowth, including anticonvulsants (such as phenytoin), immunosuppressants (like cyclosporine), and calcium channel blockers (such as nifedipine). Genetic predisposition or hereditary factors can play a key role in some people, leading to conditions such as hereditary gingival fibromatosis (2). A surge in sex steroid hormones, mainly testosterone in males and estrogen in females, triggers significant changes in physical appearance and behaviour during puberty. In females, estrogen and progesterone production increase during puberty and remain relatively constant throughout the normal reproductive phase. Within the gingiva, fibroblast and keratinocyte cell division, growth, and differentiation can be impacted by these steroid hormones. Progesterone primarily promotes the production of inflammatory mediators, while estrogen contributes to alterations in blood vessels and elevates the occurrence of certain species, such as Capnocytophaga and Prevotella intermedia (3). Further exacerbating gingival inflammation can be the complicated relationship between periodontal bacteria and sex hormones. Bacteroides melanogenicus (now Prevotella intermedia) uses progesterone and estrogen as vitamin K substitutes, leading to increased bacterial growth during hormonal elevations. The transition in the microbiota is a contributing factor to the higher incidence of gingivitis observed during pregnancy and puberty (4). Beyond physiological hormonal cycle fluctuations, such as those associated with growth hormone and pregnancy, pathological endocrine conditions may further impact periodontal tissue responses. Polycystic Ovary Syndrome (PCOS), one of the most common endocrine disorders affecting females of reproductive age, is characterised by hyperandrogenism, ovulatory dysfunction, and insulin resistance. These persistent hormonal and metabolic changes may induce a chronic, low-grade inflammatory state that can aggravate gingival inflammation even when plaque formation is minimal (5). PCOS is a reproductive disorder affecting the ovaries, with a prevalence ranging from 4% to 20%. Symptoms typically emerge between the ages of 18 and 39 years, but diagnosis and treatment are often delayed, leaving many patients undiagnosed (6). According to research showing a bidirectional association between PCOS and periodontal disease, women with PCOS may have elevated inflammatory markers, affected subgingival microbiota, and higher gingival bleeding when compared to controls who are systemically healthy. An increased risk of inflammatory gingival enlargement can arise from insulin resistance and elevated circulating androgens, which may also increase gingival vascular permeability and alter the host immune response (7). Therefore, it is essential to consider underlying endocrine disorders like PCOS as well as physiological pubertal hormonal changes while diagnosing adolescent females who have increased gingival growth. Clinically, puberty gingivitis is defined by increased gingival bleeding during puberty and an initial phase of robust inflammation of the marginal gingiva and, via direct progression, of the attached gingiva, including the interdental papillae. Mostly, the lingual surfaces are located on the facial surfaces and are largely unaffected by this gingival expansion (8).

## Case Report

**Figure 1:**
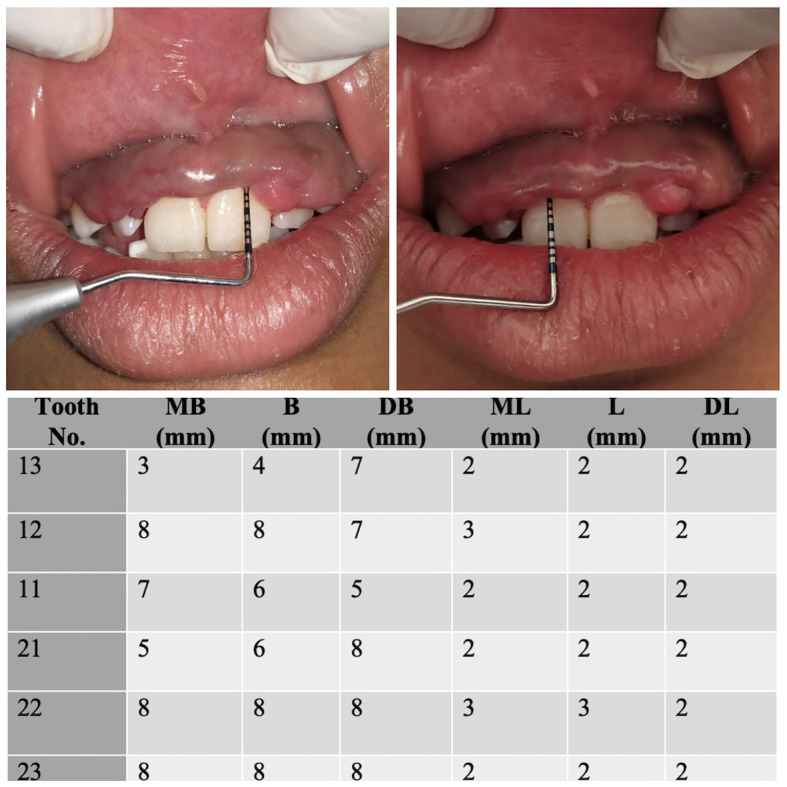
Assessment of periodontal pocket depth using the UNC 15 probe along with periodontal charting.

A 17-year-old girl presented to the Department of Periodontology and Implantology, having a chief complaint of spontaneous bleeding in the mandibular and anterior maxillary areas, along with swollen gums for one year. The patient was apparently healthy one year prior; however, she then observed a subtle rise in gingival swelling and mild bleeding. As the disease progressed, there was considerable gingival overgrowth and spontaneous bleeding during brushing and chewing. There was no previous history of systemic disease or drug intake. During intraoral examination, diffuse gingival hypertrophy affecting the marginal, interdental, and attached gingiva in the maxillary arch extending from tooth #13 to #23 was recognised. The gingiva appeared bluish-red, soft, and oedematous. Grade III gingival enlargement was observed in relation to #12, #22, and #23, while #11 and #21 presented Grade II enlargement (9). Spontaneous bleeding on probing was observed, with a Periodontal probing pocket depth and no clinical attachment loss (Fig. 1), confirming the presence of pseudo pockets.

1

**Figure 2:**
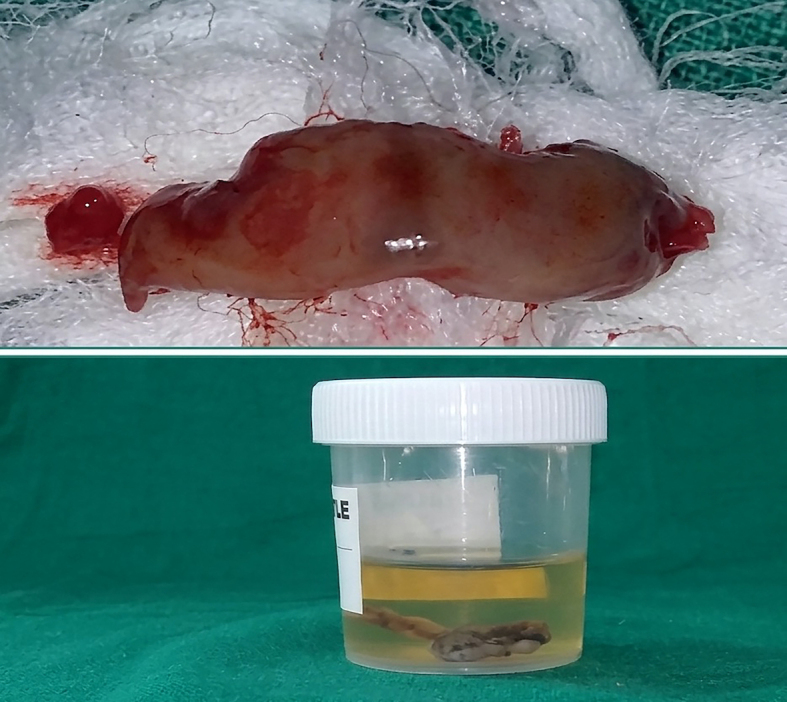
Excised soft tissue specimen.

An OHI-S (Greene and Vermillion, 1964) score of 2 indicated fair oral hygiene, which did not correlate with the intensity of the enlargement (10). Taking into consideration the duration of symptoms and the degree of gingival growth, a thorough menstrual and endocrine history was recorded. The patient complained of sudden weight gain, sporadic acne flare-ups, and irregular menstrual cycles since menarche. In light of these findings, she was encouraged to have a medical evaluation. Upon recall, evaluation revealed elevated serum testosterone levels, increased LH/FSH ratio (>2:1), and raised fasting insulin levels, consistent with hyperandrogenism and insulin resistance. Pelvic ultrasonography demonstrated polycystic ovarian morphology. The medical evaluation suggested features consistent with Polycystic Ovary Syndrome (PCOS). Based on the menstrual history and clinical and laboratory findings, a provisional diagnosis of inflammatory gingival enlargement modified by hormonal imbalance with a possible systemic endocrine association was established, and a holistic treatment approach was planned. After explaining the treatment plan to the patient and obtaining informed consent, non-surgical periodontal therapy was performed. The patient was instructed to maintain adequate oral hygiene, and chlorhexidine (0.12%) mouthwash was prescribed twice a day. The patient was simultaneously referred to a gynaecologist/endocrinologist for management of PCOS, including lifestyle modification, dietary counselling, and hormonal regulation therapy. However, no marked difference was observed after 15 days, even with improved oral hygiene, to completely remove the thickened gingival tissues and recontour the gingiva to restore its normal architecture. Therefore, surgical intervention consisting of gingivectomy and gingivoplasty was planned. - Surgical Procedure A comprehensive haematological evaluation was conducted on the day of the surgical procedure, and all variables were within normal ranges. After a patch test, local anaesthesia with adrenaline (1: 80,000) was administered. The Krane-Kaplan pocket marker was used to mark bleeding points. A scalpel with blade #15 was used to make a continuous external bevel incision, ensuring an angle of around 45° towards the tooth surface. The excised gingival tissue was carefully removed using a sterile Gracey curette to ensure complete elimination of the overgrowth, and the specimen was kept in a biopsy bottle for excisional biopsy (Fig. 2).

2

**Figure 3:**
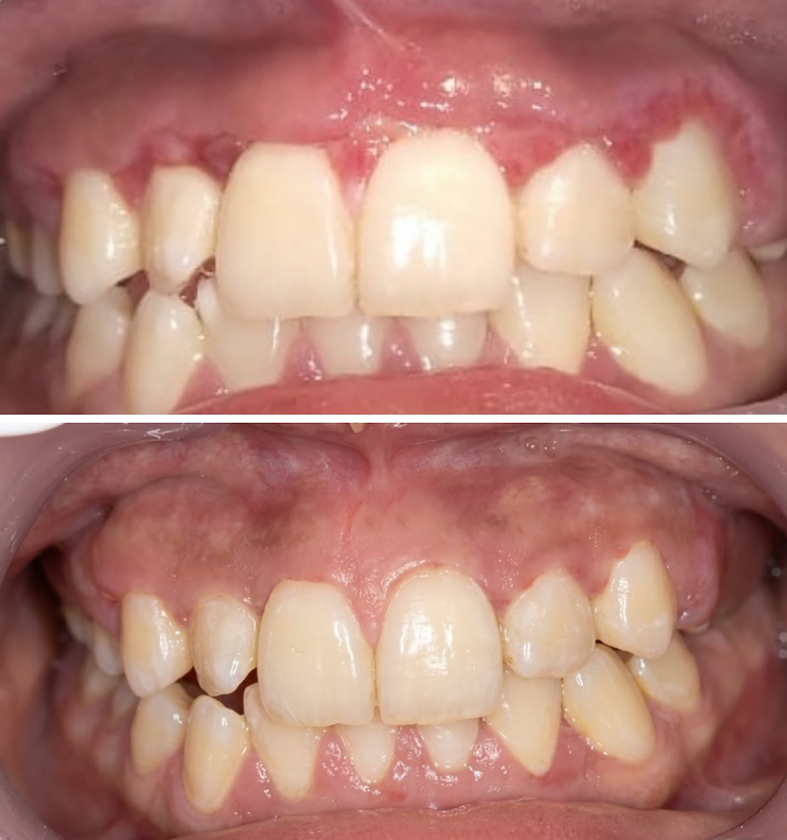
Post-operative analysis a) after 7 days, b) after 2 months.

As a coagulating adjunct, electrocautery (ART Electron, BonART Co. Ltd.) was used to achieve hemostasias. The gingiva was then recontoured and reshaped with gingivoplasty, and the surgical site was protected with a periodontal dressing (Coe-Pak) to encase the wound and promote healing. The patient was given the post-operative instructions and prescribed prophylactic medication for 5 days. Follow-up visits were scheduled at 7 days and 2 months, and the outcomes were satisfactory (Fig. 3).

3

**Figure 4:**
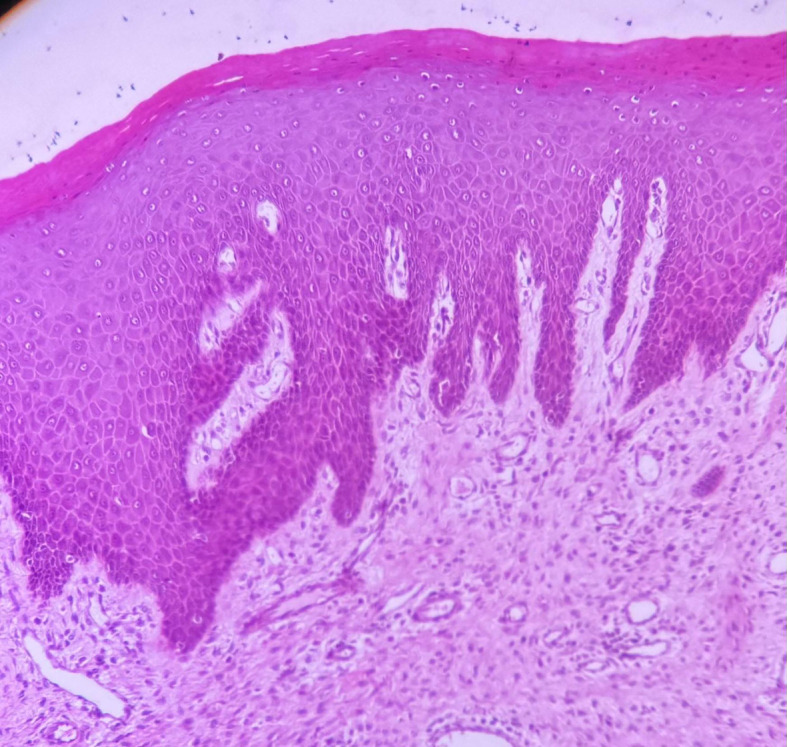
Photomicrograph of gingival tissue with Polycystic Ovary Syndrome. (Hematoxylin and eosin stain; original magnification ×200).

- Histopathology Histomorphology features of the excised tissue revealed para keratinized stratified squamous epithelium exhibiting acanthosis and elongated rete ridges , marked increase in collagen deposition, active fibroblastic proliferation, reduced elastic fibres, increased vascularity with dilated capillaries and a moderate inflammatory infiltrate. These findings are suggestive of fibrotic pattern of inflammatory gingival enlargement with hormonally influenced changes (Fig. 4).

4

## Discussion

Several factors, including systemic diseases, medications, hormonal fluctuations, and local inflammatory factors, can cause gingival enlargement. The 2017 World Workshop classification categorised gingival enlargement associated with puberty as a gingival disease caused by dental plaque altered by systemic factors, specifically hormonal changes (1). However, even with relatively small plaque deposits, hormonal shifts during puberty enhance gingival inflammatory reactions by altering host immune responses and increasing gingival vascular permeability (11). Sex steroid hormones, such as estrogen and progesterone, affect the gingival microenvironment by promoting vasodilation, increasing capillary fragility, and altering subgingival bacteria, thereby amplifying inflammatory reactions and promoting gingival overgrowth. Puberty-associated enlargement often manifests as generalised gingival enlargement involving the anterior segments and interdental papillae, which can be explained by these hormonal effects (8 , 12). In recent years, substantial consideration has been given to endocrine disorders, including Polycystic Ovary Syndrome (PCOS), as prospective influencing factors in periodontal diseases. PCOS is identified by hyperandrogenism, chronic anovulation and insulin resistance, which contribute to pervasive hormonal imbalance besides the physiological changes found during puberty. These hormonal changes may further increase gingival swelling and inflammation in vulnerable teenage and young female individuals (13). Evidence suggests that hormonal changes during puberty may enhance the gingival inflammatory response to plaque accumulation. Studies have demonstrated that non-surgical periodontal therapy combined with meticulous plaque control can result in significant regression of puberty-associated gingival enlargement, indicating the presence of a reversible inflammatory component (14). PCOS, through pathways involving reactive oxygen species, insulin resistance, AGE, and hormonal levels, could affect periodontal conditions already influenced by plaque, establishing a link between systemic hormonal disorders and periodontal health (15). In patients with PCOS, raised circulating androgens and changed estrogen-progesterone proportions might alter gingival fibroblast production, collagen breakdown, and inflammatory mediator expression in a reminiscent manner, but potentially more enduring than puberty-associated hormonal alterations. Furthermore, insulin resistance, which is generally associated with PCOS, promotes a pro-inflammatory state that may increase periodontal tissue susceptibility to plaque-induced inflammation (16). The results of the current case are consistent with past studies, indicating that hormonal changes contribute to exaggerating tissue response, while plaque accumulation remains the main initiating factor. Differential diagnosis plays an important role in the assessment of gingival enlargement, especially in distinguishing hormone-related enlargement from drug-induced gingival overgrowth, idiopathic gingival fibromatosis, leukemic enlargement, and systemic-related gingival lesions. Although drug-induced enlargement, like phenytoin-associated gingival overgrowth, usually has a fibrotic consistency and may require surgical management in addition to controlling plaque (2). Gingival enlargement in conjunction with PCOS may physiologically appear like puberty gingivitis, but may exhibit a more persistent inflammatory condition due to chronic endocrine imbalance. Recognition of these associations is crucial since periodontal therapy alone might not assure long-term stability, regardless of whether systemic hormone management is concurrently addressed in coordination with a gynaecologist or endocrinologist (13). The management of gingival enlargement associated with puberty includes eradicating local etiologic factors, educating patients, reinforcing meticulous oral hygiene, and seeking periodic professional maintenance. Gingivectomy and gingivoplasty are surgical procedures that should be performed when functional and aesthetic issues persist or when fibrotic enlargement persists after inflammation has been adequately controlled. Long-term follow-up is essential because recurrence may occur during periods of continued hormonal fluctuation. In cases where PCOS is established, holistic care comprising lifestyle acclimatisation, medical supervision of insulin resistance, and hormone therapy may indirectly enhance periodontal results by controlling systemic inflammatory and endocrine parameters.

## Conclusions

Overall, contemporary evidence suggests that gingival enlargement associated with puberty is a complex, hormone-modified inflammatory disease affected by endocrine changes. Emerging research furthermore indicates that systemic endocrine abnormalities such as PCOS may operate as additional modifying variables, enhancing gingival inflammatory responses in teenage and young adult females. This surgical case report illustrates that successful management and long-term periodontal health are largely determined by early diagnosis, preventive periodontal care, patient compliance, and, when indicated, interdisciplinary medical collaboration.
